# Xylitol Affects the Intestinal Microbiota and Metabolism of Daidzein in Adult Male Mice

**DOI:** 10.3390/ijms141223993

**Published:** 2013-12-10

**Authors:** Motoi Tamura, Chigusa Hoshi, Sachiko Hori

**Affiliations:** National Food Research Institute, National Agriculture and Food Research Organization, Tsukuba, Ibaraki 305-8642, Japan; E-Mails: chig3@affrc.go.jp (C.H.); vets@affrc.go.jp (S.H.)

**Keywords:** xylitol, equol, daidzein, mice, intestinal microbiota

## Abstract

This study examined the effects of xylitol on mouse intestinal microbiota and urinary isoflavonoids. Xylitol is classified as a sugar alcohol and used as a food additive. The intestinal microbiota seems to play an important role in isoflavone metabolism. Xylitol feeding appears to affect the gut microbiota. We hypothesized that dietary xylitol changes intestinal microbiota and, therefore, the metabolism of isoflavonoids in mice. Male mice were randomly divided into two groups: those fed a 0.05% daidzein with 5% xylitol diet (XD group) and those fed a 0.05% daidzein-containing control diet (CD group) for 28 days. Plasma total cholesterol concentrations were significantly lower in the XD group than in the CD group (*p* < 0.05). Urinary amounts of equol were significantly higher in the XD group than in the CD group (*p* < 0.05). The fecal lipid contents (% dry weight) were significantly greater in the XD group than in the CD group (*p* < 0.01). The cecal microbiota differed between the two dietary groups. The occupation ratios of *Bacteroides* were significantly greater in the CD than in the XD group (*p* < 0.05). This study suggests that xylitol has the potential to affect the metabolism of daidzein by altering the metabolic activity of the intestinal microbiota and/or gut environment. Given that equol affects bone health, dietary xylitol plus isoflavonoids may exert a favorable effect on bone health.

## 1. Introduction

Xylitol is classified as a sugar alcohol and used as a food additive and in medications. It has been reported that xylitol is a suitable component of a diabetic diet [1] and intake of xylitol may be beneficial in preventing the development of obesity and metabolic abnormalities in rats with diet-induced obesity [2].

It has been suggested that xylitol is a good source of energy of the rats treated with CCl_4_ (carbon tetrachloride) because xylitol is more efficiently oxidized to CO_2_ than glucose in the livers treated with CC1_4_[3]. It has been reported that xylitol restricted the ovariectomy-induced reduction in bone density, in bone ash weight and in concentrations of humeral calcium and phosphorus in ovariectomized (ovx) rats [4]. Furthermore, trabecular bone loss in ovx rats was significantly decreased by dietary xylitol [4]. It has been further reported that xylitol played a protective role against osteoporosis [5]. Xylitol administration has shown improvements in bone biochemical properties [6] and retards the ovariectomy-induced increase of bone turnover in rats [7]. The stimulation of calcium (Ca) absorption in male rats after feeding diets containing xylitol has been elucidated in [8]. Thus, xylitol has favorable effects on bone metabolism.

Isoflavones are a class of phytoestrogens because they bind to the estrogen receptors, albeit weakly compared to endogenous estrogens [9]. It has been suggested that the preventive effect of daidzin, genistin and glycitin significantly prevented bone loss in ovx rats at a dose of 50 mg/kg/day, like estrone [10]. It has been reported that genistein was slightly lower in estrogenic potency than equol with an EC_50_ of 0.5 μM but significantly more potent than the structurally similar compounds daidzein and biochanin A (*p* < 0.01) [11].

**Figure 1.**
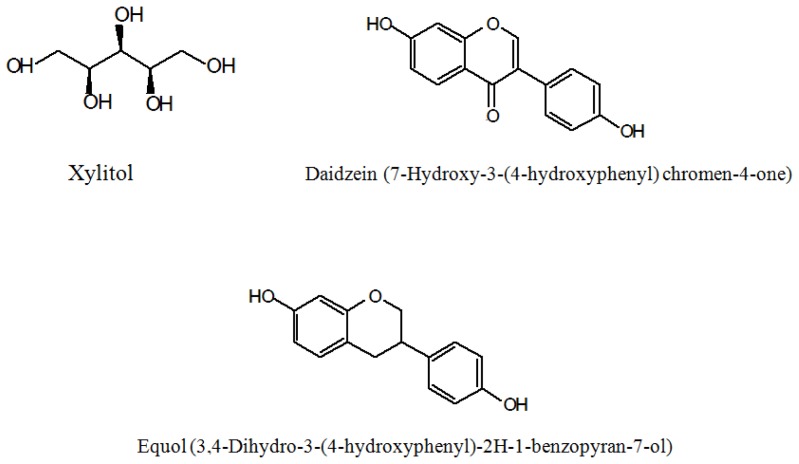
Chemical structure of xylitol, daidzein and equol.

Human gastrointestinal bacteria seem to play an important role in isoflavone metabolism [12–16]. Equol is a metabolite of daidzein produced by intestinal microbiota [17]. The chemical structure of daidzein, equol and xylitol is shown in Figure 1. It has also been suggested that the ability to produce equol or equol itself, is closely related to a lower prevalence of prostate cancer [18]. Equol is an important bacterial metabolite in the gut. However, interindividual variations in equol production have been identified. Only 30% to 50% of humans are equol producers [19].

Recently, much attention has been focused on the relationship between intestinal microbiota and obesity. Studies on human volunteers have revealed that obesity is associated with changes in the relative abundance of the two dominant bacterial divisions, the Bacteroidetes and the Firmicutes [20].

On the other hand, xylitol feeding caused a clear shift in the rodent fecal microbial population from Gram-negative to Gram-positive bacteria [21]. Xylitol affects the fecal microbiota [21]. Xylitol feeding seems to affect the gut microbiota.

We tested the hypothesis that dietary xylitol changes the metabolism of isoflavonoids and intestinal microbiota in mice.

## 2. Results and Discussion

### 2.1. General Observations

No significant differences were observed between the control-daidzein (CD) and xylotol-daidzein (XD) groups in final body weight (g) CD (32.2 ± 1.1) and XD (34.4 ± 1.0), food consumption CD (4.26 ± 0.02) and XD (4.27 ± 0.02), visceral fat (g) CD (1.44 ± 0.24) and XD (1.49 ± 0.25), amount of feces (g/day) CD (0.34 ± 0.01) and XD (0.34 ± 0.01) or liver weight (g) CD (1.40 ± 0.05) and XD (1.58 ± 0.09). The cecal contents were significantly higher in the XD group (0.26 ± 0.02) than in the CD group (0.12 ± 0.01) (*p* < 0.01).

### 2.2. Urinary Isoflavonoids

**Figure 2.**
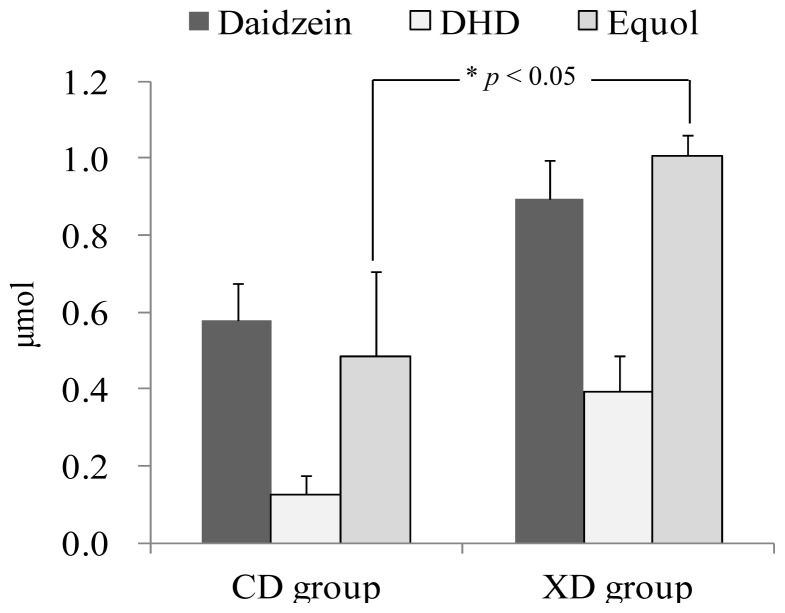
Amounts of urinary isoflavonoids of mice in the control diet (CD) group and the xylitol diet (XD) group. Enzymatic hydrolysis of the urinary isoflavone glucuronides was carried out with β-glucuronidase/arylsulfatase from *Helix pomatia*. We measured the urinary isoflavonoids as aglycones. Values are means ± SE (*n* = 7). * Significantly different (*p* < 0.05) from the CD group. The data were analyzed using the Student’s *t*-test (equol). Statistical significance was reached with a *p* value of less than 0.05.

**Figure 3.**
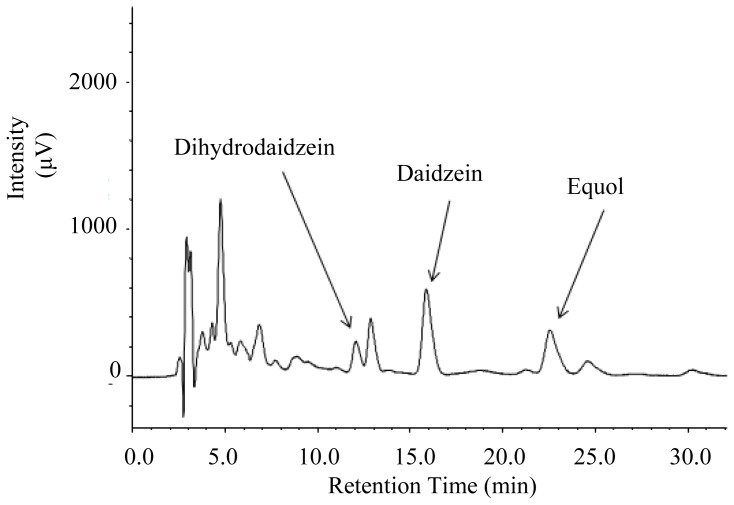
HPLC chromatogram of daidzein, dihydrodaidzein and equol obtained from urine of a mouse fed the XD diet at 280 nm. Peak identity was confirmed by rechromatography with the authentic compounds.

**Figure 4.**
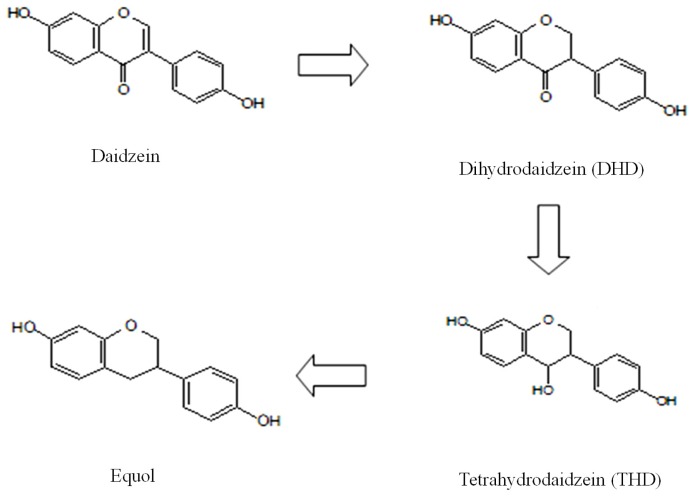
Proposed pathway for daidzein reduction by intestinal microbiota [22].

Xylitol affected the amount of daidzein and its metabolites found in the urine (Figure 2). An HPLC chromatogram obtained from urine of a mouse fed the XD diet is shown in Figure 3. In our results, significant amounts of DHD (dihydrodaidzein), which is a precursor of equol, were excreted in the urine. The proposed pathway for daidzein reduction by intestinal microbiota is shown in Figure 4.

Average urinary amounts of daidzein and dihydrodaidzein (DHD) tended to be higher in the XD group than in the CD group. It has been reported that rat intestinal microbiota rapidly metabolized daidzein to aliphatic compounds that could not be detected by HPLC or mass spectral analysis [23]. Degradation activity of intestinal microbiota against daidzein might have differed between the two groups.

The urinary amounts of equol were significantly higher in the XD group than in the CD group (*p* < 0.05) (Figure 2). Xylitol was characterized by a significantly increased production of short-chain fatty acids (SCFA), particularly the concentration of butyrate [24]. The addition of butyrate increased the equol production in equol-producing bacteria [25]. It has been reported that butyrate increased the conversion ratio of daidzein to equol in equol-producing bacteria [25]. Our results suggest that dietary xylitol might induce equol production by stimulating butyrate-producing bacteria in the fecal microbiota of the mice.

On the other hand, xylitol decreased the rate of gastric emptying but concomitantly accelerated intestinal transit compared with glucose [26]. Thus, xylitol administration might alter the gut environment and metabolism of isoflavonoids. Our results suggest that dietary xylitol has the potential to affect the metabolism of equol by altering the metabolic activity of the intestinal microbiota and/or digestion and absorption of isoflavonoids.

It has been indicated that xylitol exerted beneficial effects on bone health [4,8,27,28]. Equol also affects the bone health [29,30]. It has been reported that a combination of dietary fructooligosaccharides and isoflavone conjugates increases femoral bone mineral density and equol production in ovx mice [30]. It has been suggested that 10 mg/day of equol supplementation contributes to bone health in non-equol-producing postmenopausal women without adverse effects [31]. Dietary xylitol plus isoflavonoids may exert a synergic effect on bone health, resulting in the prevention of the osteoporosis.

### 2.3. Amount of Fecal Lipid Contents

**Figure 5.**
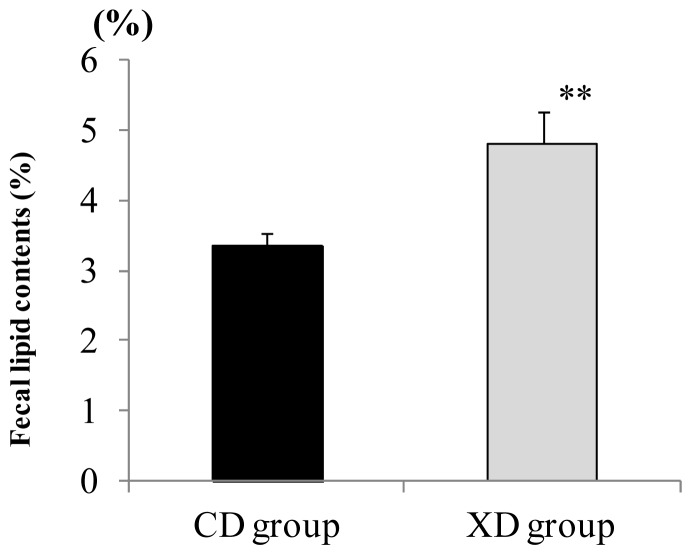
The fecal lipid contents (% dry weight) sampled on the final days of the experiment were significantly greater in the XD group than in the CD group (*p* < 0.01) Values are means ± SE (*n* = 7). The data were analyzed using the Student’s *t*-test analysis. ** Significantly different (*p* < 0.01) from the CD group.

The XD diet significantly affected the fecal lipid contents. The fecal lipid contents (% dry weight) of feces sampled on the final days of the experiment were significantly greater in the XD group than in the CD group (*p* < 0.01), as shown in Figure 5.

Xylitol seems to affect gut function. It has been reported that after ingestion of 25 g xylitol, gastric emptying was markedly prolonged in human volunteers [32]. Prolonged gastric emptying by xylitol might reduce the absorption of lipids, resulting in the increase of the fecal lipid contents in the XD group.

### 2.4. Plasma Total Cholesterol, Triglycerides, and Phospholipids

**Figure 6.**
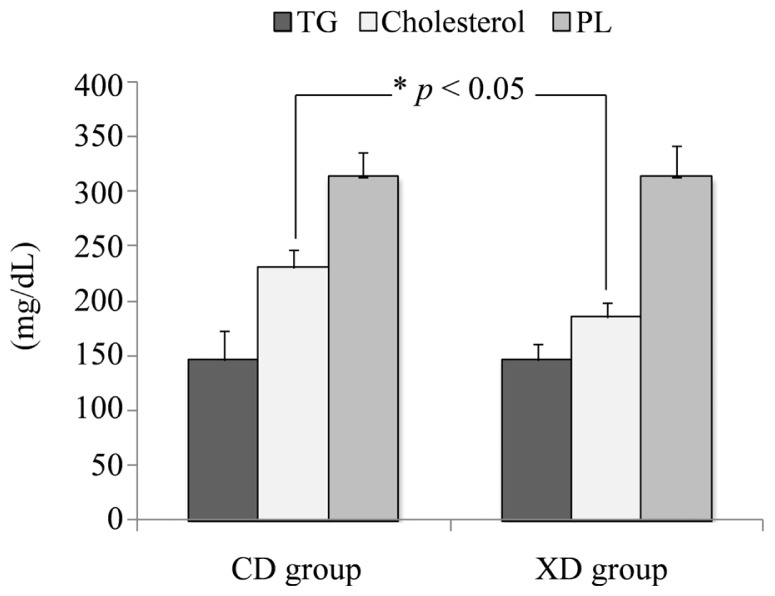
Plasma total cholesterol, triglycerides (TG) and phospholipids (PL) concentrations of mice in the xylitol-daidzein (XD) and control-daidzein (CD) groups. Values are means ± SE (*n* = 7). The data were analyzed using the Student’s *t*-test analysis (*p* < 0.05). * Significantly different (p < 0.05) from the CD group.

The plasma was separated from whole blood by centrifugation and used for analysis of plasma triglycerides, total cholesterol and phospholipids. Plasma lipids are shown in Figure 6. Plasma cholesterol concentrations were modestly lower in the XD (185.2 ± 13.0 mg/dL) than in the CD (230.8 ± 16.2 mg/dL) group (*p* < 0.05). No significant differences in the plasma triglyceride (TG) concentrations (CD, 146.6 ± 26.6 mg/dL; XD, 146.8 ± 14.0 mg/dL) or plasma phospholipids (PL) concentrations (CD, 313.7 ± 22.3 mg/dL; XD, 313.6 ± 28.9 mg/dL) were observed between the two groups.

It was reported that an isoflavone aglycone-rich extract without soy protein attenuated atherosclerosis development in cholesterol-fed rabbits [33]. Dietary daidzein contained in the experimental diet affected the plasma lipids in both the CD and XD groups. However, plasma total cholesterol concentrations were significantly lower in the XD group than in the CD group. The contribution of daidzein was not evident at the plasma cholesterol level. It has been reported that xylitol-fed rats had significantly lower serum total cholesterol levels than control rats [34,35]. Dietary xylitol might have potent cholesterol-lowering effect in mice. However, no significant differences in serum triglycerides were observed between the xylitol-fed rat and control rats [34]. Though our experimental diet contained 0.05% daidzein, these results agree with ours. Plasma total cholesterol concentrations were significantly lower in the XD group than in the CD group. These results suggest that dietary xylitol has modest effects on lipid absorption in mice.

### 2.5. Effects of Diet on Cecal Microbiota of Mice

**Figure 7.**
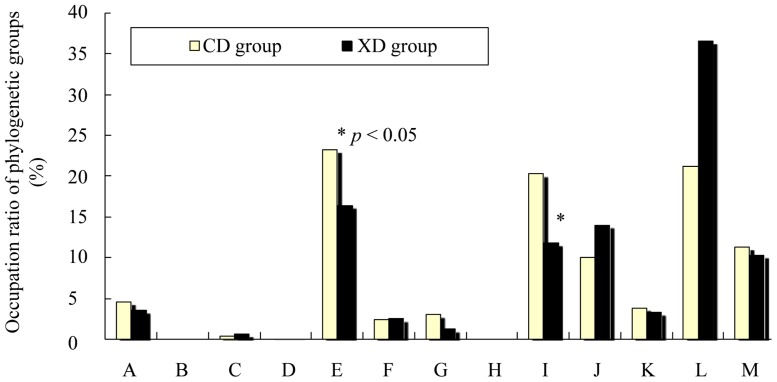
Composition of cecal intestinal microbiota of mice in the CD and XD groups. OTUs (operational taxonomic units), which correspond to either T-RFs (terminal restriction fragments) or T-RF clusters, detected by T-RFLP (terminal restriction fragment length polymorphism) analysis. Values are means ± SE (*n* = 7). * Significantly different (*p* < 0.05) from the CD group. Data were analyzed using the Student’s *t*-test analysis. Letters correspond to the following phylogenetic bacterial groups: (**A**) *Bacteroides*, *Clostridium* cluster IV (OTUs 370); (**B**) *Clostridium* cluster IV (OTUs 168, 749); (**C**) *Clostridium* cluster IX, *Megamonas* (OTUs 110); (**D**) *Clostridium* cluster XI (OTUs 338); (**E**) *Clostridium* subcluster XIVa (OTUs 106, 494, 505, 517, 754, 955, 990); (**F**) *Clostridium* cluster XI, *Clostridium* subcluster XIVa (OTUs 919); (**G**) *Clostridium* subcluster XIVa, *Enterobacteriales* (OTUs 940); (**H**) *Clostridium* cluster XVIII (OTUs 423, 650); (**I**) *Bacteroides* (OTUs 469, 853); (**J**) *Bifidobacterium* (OTUs 124); (**K**) *Lactobacillales* (OTUs 332, 520, 657); (**L**) *Prevotella* (OTUs 137, 317); and (**M**) others.

The compositions of the phylogenetic groups of cecal microbiota differed between the two dietary groups (Figure 7). The predominant operational taxonomic units (OTUs) [36], which correspond to either T-RFs (terminal restriction fragments) or T-RF clusters, were detected in the T-RFLP (terminal restriction fragment length polymorphism) profiles and used to identify phylogenetic groups of intestinal microbiota [37,38].

The occupation ratios of *Bacteroides* [operational taxonomic units (OTUs) 469, 853] were significantly greater in the CD than in the XD group (*p* < 0.05). Dietary xylitol-feeding may reduce *Bacteroides* (OTUs 469, 853). *Bacteroides* belongs to Gram-negative bacteria.

It has been shown that xylitol feeding caused a clear shift in rodent fecal microbial populations from Gram-negative to Gram-positive bacteria [21]. In human volunteers a similar shift was observed even after a single 30-g oral dose of xylitol [21].

It has been confirmed that human intestinal microbiota predominantly consists of members of approximately 10 phylogenetic bacterial groups and that these bacterial groups can be distinguished by the T-RFLP system developed by Nagashima *et al.* [37,38]. We used this Nagashima method [37,38] although it cannot completely distinguish between Gram-negative and Gram-positive bacteria. However, in our results, the occupation ratios of *Bifidobacterium* tended to increase in the XD group. *Bifidobacterium* belongs to Gram-positive bacteria.

The occupation ratios of *Prevotella* tended to increase in the XD group. There are few reports with respect to the effects of dietary xylitol on *Prevotella*. We cannot explain this phenomenon.

Some diets affect the composition of intestinal microbiota [39]. It has been reported that diet affects the microbiota in terms of both structure and gene expression [40]. Switching from a low-fat, plant polysaccharide-rich diet to a high-fat, high-sugar “Western” diet shifted the population of the microbiota within a single day, changed the representation of metabolic pathways in the microbiome, and altered microbiome gene expression [40]. It has been reported that dietary fat significantly affects intestinal microbiota [40,41]. In our result, the XD diet significantly affected the fecal lipid contents. Differences in lipid concentrations of the gut also might affect the composition and/or metabolic activities of intestinal microbiota.

A limitation of our study was that we could not identify what kind of intestinal bacteria were stimulated by the supplementation of dietary xylitol *in vitro.* Further studies are needed to clarify these effects.

## 3. Experimental Section

### 3.1. Materials

Daidzein and equol were purchased from LC Laboratories (Woburn, MA, USA). Dihydrodaidzein was purchased from Toronto Research Chemicals, Inc. (North York, ON, Canada). β-Glucuronidase type H-5 was obtained from Sigma (St. Louis, MO, USA). Xylitol was purchased from Wako Pure Chemical Industries, Ltd. (Osaka, Japan).

### 3.2. Treatment of Animals

Male Crj: CD-1 (ICR) mice (6 weeks old) were purchased from Charles River Japan, Inc. (Kanagawa, Japan). All mice were specific pathogen-free (SPF) and were housed in conventional conditions in our laboratory. The mice were randomly divided into two groups of seven animals each. The animals were housed individually in suspended stainless-steel cages with wire mesh bottoms, in a room kept at 24 ± 0.5 °C and a relative humidity of 65%, with 12 h periods of light and dark. Mice were fed an AIN-93M diet [42] for 9 days. After 9 days, the diet was replaced with a 0.05% daidzein (CD) diet (as control diet) or 0.05% daidzein-5% xylitol (XD) diet, for 28 days. After 21 days from the start of the experimental diet feeding, all animals were moved to individual metabolic cages (Tecniplast S.P.A., Buguggiate, Italy). Urine was collected from all mice for 45 h. Urinary amounts of isoflavonoids were measured. The purified diet and water were provided *ad libitum*. Table 1 presents the composition of each diet. Body weight and food consumption were measured during the experiment. Feces were collected during the experiment in a metabolic cage. Feces were dried with a freeze dryer (FD-1000; Tokyo Rikakikai Co., Ltd., Tokyo, Japan) for 24 h. The trap cooling temperature was −45 °C. After the experimental diet feeding period, the mice were anesthetized by 30 mg/kg of intraperitoneal injection of pentobarbital sodium and blood samples were taken from the abdominal aorta and placed in heparinized tubes. All mice were euthanized by CO_2_. The plasma was separated from whole blood by centrifugation and stored at −80 °C until analysis of plasma lipids. The liver, visceral fat, and cecal contents were collected. Cecal contents were stored at −80 °C until T-RFLP analysis of intestinal microbiota. The liver samples and visceral fat were weighed. All procedures involving mice in this study were approved by the Animal Care Committee of the National Food Research Institute (Tsukuba, Japan), in accordance with the “Guidelines for Animal Care and Experimentation” of the National Food Research Institute, National Agriculture and Food Research Organization (NARO, Tsukuba, Japan).

### 3.3. Measurement of Plasma Cholesterol, Triglycerides, and Phospholipids

The following tests were performed with kits obtained from Wako Pure Chemical Industries Ltd. (Osaka, Japan). Total plasma cholesterol concentrations were measured using a cholesterol E-test kit based on cholesterol oxidase [43]. Plasma triglyceride concentrations were measured using a triglyceride E-test kit based on the glycerol-3-phosphate oxidase method [44]. Plasma phospholipid concentrations were measured using a phospholipid C-test kit based on the choline oxidase method [45].

### 3.4. Measurements of Fecal Weight and Fecal Lipid Extraction

All feces were collected during the experiment in metabolic cage. Weights of feces were measured. Feces were then dried with a freeze dryer (FD-1000, Tokyo Rikakikai Co., Ltd., Tokyo, Japan) for 24 h. The trap cooling temperature was −45 °C. After drying, weights of freeze-dried feces were measured. Feces were milled with a food mill (TML17; TESCOM Co., Ltd., Tokyo, Japan) for 30 s. Fecal lipids were extracted from the fecal powder by the Bligh and Dyer method [46]. A milled feces (100 mg) was added to 1 mL of 0.1 M sodium acetate buffer (pH 5.0). 3.75 mL of chloroform:methanol (1:2 *v/v*) was added to the sample. All samples were vortexed for 120 s. Next, 1.25 mL of chloroform was added to the sample. All samples were vortexed for 60 s. 1.25 mL of dH_2_O was added to the sample. All samples were vortexed for 60 s and centrifuged at 3000 rpm for 10 min. The organic lower phase was removed using a Pasteur pipette and transferred to a glass test tube. The organic phase was evaporated under a gentle stream of dry nitrogen. The dried extracts were weighed.

### 3.5. Analysis of Urinary Isoflavonoids

An enzymatic hydrolysis of the isoflavone glucuronides was used for the total isoflavone content determination in the urine samples. A total of 200 μL of urine was added to 200 μL of β-glucuronidase H-5 solution (35 mg/mL; Sigma) in 0.2 M sodium acetate buffer (pH 5.0). Next, the mixture was incubated at 37 °C in a water bath for 3 h, followed by treatment with 400 μL of ethyl acetate, vortexing for 30 s, and centrifugation at 5000× *g* for 10 min at 4 °C. The supernatants were transferred to an eggplant-type flask. The same volume as that used in the first extraction of ethyl acetate was added to the sediment, and the procedure was repeated. The supernatants from both extractions were pooled in the eggplant-type flask and evaporated completely using a rotary evaporator. The sample was then dissolved in 400 μL of 80% methanol and filtered through a 0.2-μm filter. Filtrates were used for HPLC analysis. For HPLC analysis, 20 μL of each preparation were injected into a 250 × 4.6-mm Capcell Pak MG C18 5-μm column (Shiseido, Tokyo, Japan). To detect isoflavonoids, a photodiode array detector (MD-1515; JASCO, Co., Ltd., Tokyo, Japan) was used to monitor the spectral data from 200 to 400 nm for each peak. To measure the isoflavonoids, daidzein, DHD and equol were used as standard samples. The spectral data at 254 nm was used to quantify daidzein (*t*_R_ 15.8 min) and the spectral data at 280 nm was used to quantify equol (*t*_R_ 22.4 min) and DHD (*t*_R_ 12.0 min). The mobile phase consisted of methanol/acetic acid/water (35:5:60, *v*/*v*/*v*). The running conditions of HPLC were a column temperature of 40 °C and a flow rate of 1 mL/min [47].3.6. DNA Extraction from Cecal Contents

DNA extractions from cecal contents were conducted according to Matsuki’s method [48]. Cecal samples (20 mg) were washed three times by suspending them in 1.0 mL of phosphate-buffered saline (PBS) and centrifuging each preparation at 14,000× *g* to remove possible PCR inhibitors. Following the third centrifugation, the cecal pellets were resuspended in a solution containing 0.2 mL of PBS and 250 μL of extraction buffer (200 mM Tris-HCl, 80 mM EDTA; pH 9.0) and 50 μL of 10% sodium dodecyl sulfate. A total of 300 mg of glass beads (diameter, 0.1 mm) and 500 μL of buffer-saturated phenol were added to the suspension, and the mixture was vortexed vigorously for 60 s using a Mini Bead*-*Beater (BioSpec Products Inc., Bartlesville, OK, USA) at a power level of 4800 rpm. Following centrifugation at 14,000× *g* for 5 min, 400 μL of the supernatant was collected. Phenol-chloroform-isoamyl alcohol extractions were then performed, and 250 μL of the supernatant was subjected to isopropanol precipitation. Finally, the DNA was suspended in 1 mL of Tris-EDTA buffer. The DNA preparation was adjusted to a final concentration of 10 μg/mL in TE and checked by 1.5% agarose gel electrophoresis.

### 3.7. PCR Conditions and Restriction Enzyme Digestion

The PCR mixture (25 μL) was composed of Ex Taq buffer (Takara Bio Inc., Otsu, Japan), 2 mM of Mg^2+^, and each deoxynucleoside triphosphate at a concentration of 200 μM. The amount of cecal DNA was 10 ng. The primers [37] used were 5′ FAM (carboxyfluorescein)-labeled 516f (5′-TGCCAGCAGCCGCGGTA-3′) and 1510r (5′-GGTTACCTTGTTACGACTT-3′) at a concentration of 0.10 μM, template DNA, and 0.625 U of DNA polymerase (TaKaRa EX Taq; Takara Bio Inc., Otsu, Japan). This process was carried out using a PCR system (Dice system; Takara Bio Inc.). Amplification was performed with one cycle at 95 °C for 15 min, followed by 30 cycles at 95 °C for 30 s, 50 °C for 30 s, 72 °C for 1 min, and finally one cycle at 72 °C for 10 min. The amplification products were subjected to gel electrophoresis in 1.5% agarose followed by ethidium bromide staining. The PCR products were purified using spin columns (QIAquick; Qiagen KK, Tokyo, Japan) according to the manufacturer’s instructions. The purified DNA was treated with 2 U of *Bsl*I (New England Biolabs, Ipswich, MA, USA) for 3 h at 55 °C [37].

### 3.8. T-RFLP (Terminal Restriction Fragment Length Polymorphism) Analysis

T-RFLP (terminal restriction fragment length polymorphism) analysis is based on PCR amplification of a target gene. The amplification is performed with one primer whose 5′ end is labeled with a fluorescent molecule. The mixture of amplicons is then subjected to a restriction reaction using a restriction enzyme. Following the restriction reaction, the mixture of fragments is separated using either capillary or polyacrylamide electrophoresis in a DNA sequencer, and the sizes of the different terminal fragments are determined by the fluorescence detector. We used this T-RFLP analysis in our experiment. The fluorescently labeled terminal restriction fragments (T-RFs) were analyzed by electrophoresis on an automated sequence analyzer (ABI PRISM 310 Genetic Analyzer; Applied Biosystems, Life Technologies Corporation, Carlsbad, CA, USA) in GeneScan mode. The restriction enzyme digestion mixture (2 μL) was mixed with 0.5 μL of size standards (MapMarker 1000; BioVentures, Inc., Murfreesboro, TN, USA) and 12 μL of deionized formamide. The mixture was denatured at 96 °C for 2 min and immediately chilled on ice. The injection time was 30 s for analysis of T-RFs from digestion with *Bsl*I. The run time was 40 min. The lengths and peak areas of T-RFs were determined with GeneMapper software (Applied Biosystems, Life Technologies Corporation). The predominant OTUs, which correspond to either T-RFs or T-RF clusters, were detected in the T-RFLP profiles and used to identify phylogenetic groups of intestinal microbiota [37,38].

### 3.9. Statistics

Data are expressed as the mean ± standard error (SE). All data were analysed using Sigma Plot 11 (Systat Software, Inc., San Jose, CA, USA). The data were analysed with the Student’s *t*-test or Mann-Whitney rank sum test. Statistical significance was reached with a *p*-value < 0.05.

## 4. Conclusions

In conclusion, plasma total cholesterol concentrations were significantly lower in the XD group than in the CD group. Urinary amounts of equol were significantly higher in the XD group than in the CD group. The fecal lipid contents (% dry weight) were significantly greater in the XD group than in the CD group. The cecal microbiota differed between the two dietary groups. The occupation ratios of *Bacteroides* (OTUs 469, 853) were significantly greater in the CD than in the XD group (*p* < 0.05). This study suggests that xylitol has the potential to affect the metabolism of daidzein by altering the metabolic activity of the intestinal microbiota and/or gut environment. Given that equol affects bone health, dietary xylitol plus isoflavonoids may exert a favorable effect on bone health.

## Figures and Tables

**Table 1.** Composition of the experimental diet.

Ingredient (g/kg Diet)	AIN-93M	CD Diet	XD Diet
Corn starch	465.692	455.692	405.692
Casein	140	140	140
α-Corn starch	155	154.5	154.5
Sucrose	100	100	100
Lard	-	50	50
Soy bean oil	40	-	-
Cellulose	50	50	50
Mineral mix (AIN-93M-Mix)	35	35	35
Vitamin mix (AIN-93-Mix)	10	10	10
l-Cystine	1.8	1.8	1.8
Tert-butylhydroquinone	0.008	0.008	0.008
Choline bitartrate	2.5	2.5	2.5
Xylitol	-	-	50
Daidzein	-	0.5	0.5
